# Chromosome-Level Assembly of the Common Lizard (*Zootoca vivipara*) Genome

**DOI:** 10.1093/gbe/evaa161

**Published:** 2020-08-24

**Authors:** Andrey A Yurchenko, Hans Recknagel, Kathryn R Elmer

**Affiliations:** Institute of Biodiversity, Animal Health and Comparative Medicine, College of Medical, Veterinary and Life Sciences, University of Glasgow, United Kingdom; Institute of Biodiversity, Animal Health and Comparative Medicine, College of Medical, Veterinary and Life Sciences, University of Glasgow, United Kingdom; Institute of Biodiversity, Animal Health and Comparative Medicine, College of Medical, Veterinary and Life Sciences, University of Glasgow, United Kingdom

**Keywords:** linkage map, lizard genome, multiple paternity, reptile genomics, Lacertidae, squamates

## Abstract

Squamate reptiles exhibit high variation in their phenotypic traits and geographical distributions and are therefore fascinating taxa for evolutionary and ecological research. However, genomic resources are very limited for this group of species, consequently inhibiting research efforts. To address this gap, we assembled a high-quality genome of the common lizard, *Zootoca vivipara* (Lacertidae), using a combination of high coverage Illumina (shotgun and mate-pair) and PacBio sequencing data, coupled with RNAseq data and genetic linkage map generation. The 1.46-Gb genome assembly has a scaffold N50 of 11.52 Mb with N50 contig size of 220.4 kb and only 2.96% gaps. A BUSCO analysis indicates that 97.7% of the single-copy Tetrapoda orthologs were recovered in the assembly. In total, 19,829 gene models were annotated to the genome using a combination of ab initio and homology-based methods. To improve the chromosome-level assembly, we generated a high-density linkage map from wild-caught families and developed a novel analytical pipeline to accommodate multiple paternity and unknown father genotypes. We successfully anchored and oriented almost 90% of the genome on 19 linkage groups. This annotated and oriented chromosome-level reference genome represents a valuable resource to facilitate evolutionary studies in squamate reptiles.

**Significance**

We generated a high quality, chromosome-level, annotated and oriented reference genome for the Eurasian common lizard, Zootoca vivipara. This species is the most broadly geographically distributed terrestrial squamate and has lineages that are either oviparous or viviparous. Therefore, this genome is a critical resource for advancing research on evolution, ecology, physiology, reproduction, and development.

## Introduction

Squamate reptiles are one of the largest group of vertebrates, with >10,000 species distributed worldwide. They have evolved extraordinary complex biological traits, such as live-bearing (Blackburn 2006; Pyron and Burbrink 2014), parthenogenesis (Neaves and Baumann 2011), and chromosomal variation (Deakin and Ezaz 2014). However, the lack of high-quality squamate genome assemblies has slowed research on understanding some of their hallmark adaptive traits.

Among squamates, the family Lacertidae, distributed across Eurasia and Africa, is the most species rich group of reptiles in Europe. Lacertids have adapted to various environments, from hot deserts to the coldest areas colonized by any reptile (Garcia-Porta et al. 2019), vary in traits such as coloration, including “paper-rock-scissors” strategies (Sinervo et al. 2007), and reproductive mode, including parthenogenic and live-bearing species (Neaves and Baumann 2011; Sites et al. 2011). One of these live-bearer—or viviparous—species is the Eurasian common lizard, *Zootoca vivipara*, a fascinating ecological and evolutionary model. It has the broadest natural range and the most northern distribution among terrestrial reptiles (Herczeg et al. 2003; Garcia-Porta et al. 2019). Inhabiting a range of altitudes, it has become a model for terrestrial ectotherm response to climate change and proximate stresses (Bestion et al. 2015, 2017; Dupoué et al. 2017, 2018). Several major intraspecific lineages have a divergence time of maximally ∼6 Myr (Cornetti et al. 2014; Horreo et al. 2018), and these strikingly include differing reproductive modes (viviparous and oviparous), associated life history traits, and reproductive physiologies (Foucart et al. 2014; Recknagel and Elmer 2019). Although sex determination and chromosomes differ across squamates (Pennell et al. 2018), the karyotype is generally conserved across lacertids (Rovatsos et al. 2016); however, *Z. vivipara* seems to be an exception showing variation in sex chromosome structure across lineages (Kupriyanova et al. 2008). However, to date no reference genome has been available.

We combined high-coverage Illumina-derived sequencing with multilayer PacBio and RNA-seq-based scaffolding to generate a high-quality genome assembly of the Eurasian common lizard, *Z. vivipara* (Lacertidae). An available genome of this lizard will facilitate studies of parity mode evolution, chromosomal architecture of sex determination, and environmental adaptations exhibited by this and other squamate reptiles.

## Materials and Methods

### Genome Biological Sample

The reference genome was constructed using a wild-caught adult female (heterogametic sex) collected from the Isle of Cumbrae, Scotland (permission of Scottish Natural Heritage 64972). This represents an exemplar from the Western Viviparous lineage (Surget-Groba et al. 2006; Recknagel et al. 2018), with a karyotype of *n* = 17 autosomes and *Z_1_Z_2_W* sex determination (Odierna et al. 1998; Kupriyanova et al. 2008). Euthanization followed Home Office protocols.

### DNA Sequencing and Quality Control

For Illumina sequencing, high molecular weight DNA was extracted from tail tissue with the Dneasy Blood and Tissue Kit (Qiagen) following the manufacturer’s protocol with additional Riboshredder and phenol–chloroform clean-up. A TruSeq PCR-free library with 350 bp insert size was generated by Edinburgh Genomics for one lane of Illumina HiSeqX sequencing. Nextera mate-pair libraries of 3–5 and 8–12 kb were generated by Liverpool Centre for Genomic Research and sequenced on one lane of HiSeq4000 at Edinburgh Genomics.

For PacBio sequencing, we used a standard phenol–chloroform isolation method (Sambrook and Russell 2006) with minimal shaking. A 20-kb insert library was generated by the Centre for Genomic Research (NBAF Liverpool) and sequenced with four cells on a PacBio Sequel.

Raw reads were checked using FastQC v0.11.5 (Andrews 2015) and trimmed using Trimmomatic v036 (Bolger et al. 2014). We applied a read error correction using QuorUM v.1.1.0 (Marçais et al. 2015) to the short-insert size (350 bp) paired-ends [PEs]. Nextera junction adapters in the long insert size mate-pairs (3–5, 8–12 kb) were removed with NxTrim v0.4.1 (O’Connell et al. 2015).

### RNA-Sequencing

Total RNA was extracted from RNAlater-preserved tissue (intestine, lungs, liver, muscle) using PureLink RNA Mini Kits (Life Technologies, Carlsbad, CA) following the protocol by Gunter et al. (2013). Libraries were prepared for each tissue separately with the Illumina TruSeq Total Stranded RNA-seq protocol by Edinburgh Genomics and sequenced on one lane of an Illumina HiSeq4000 (150 bp PEs).

### Genome Assembly

Genome size was estimated using the k-mer distribution method of SGA v0.10.15 (Simpson 2014). Contigs were assembled using the Platanus v1.2.4 assembler (Kajitani et al. 2014). Initial scaffolding was performed using the *platanus scaffold* command with all the reads excluding by-product PE and SE from mate-pair libraries. Next, the resulting scaffolds were rescaffolded with the PacBio long reads (at least 1,000 bp long to reduce the chimera rate) and the 8–12 kb mate pairs using the OPERA-LG v2.0.6 software (Gao et al. 2016) and a k-mer size = 50.

The scaffolds outputted by OPERA-LG were additionally scaffolded using AGOUTI v0.3.3 (Zhang et al. 2016), which uses RNA-seq data and splicing information. To apply the AGOUTI algorithm, we first identified coding sequences in the draft genome using AUGUSTUS v3.3 (Stanke et al. 2004) and mapped the RNA-seq reads to the genome with the BWA v0.7.15-r1140 *mem* algorithm (Li and Durbin 2009).

**Fig. 1.**
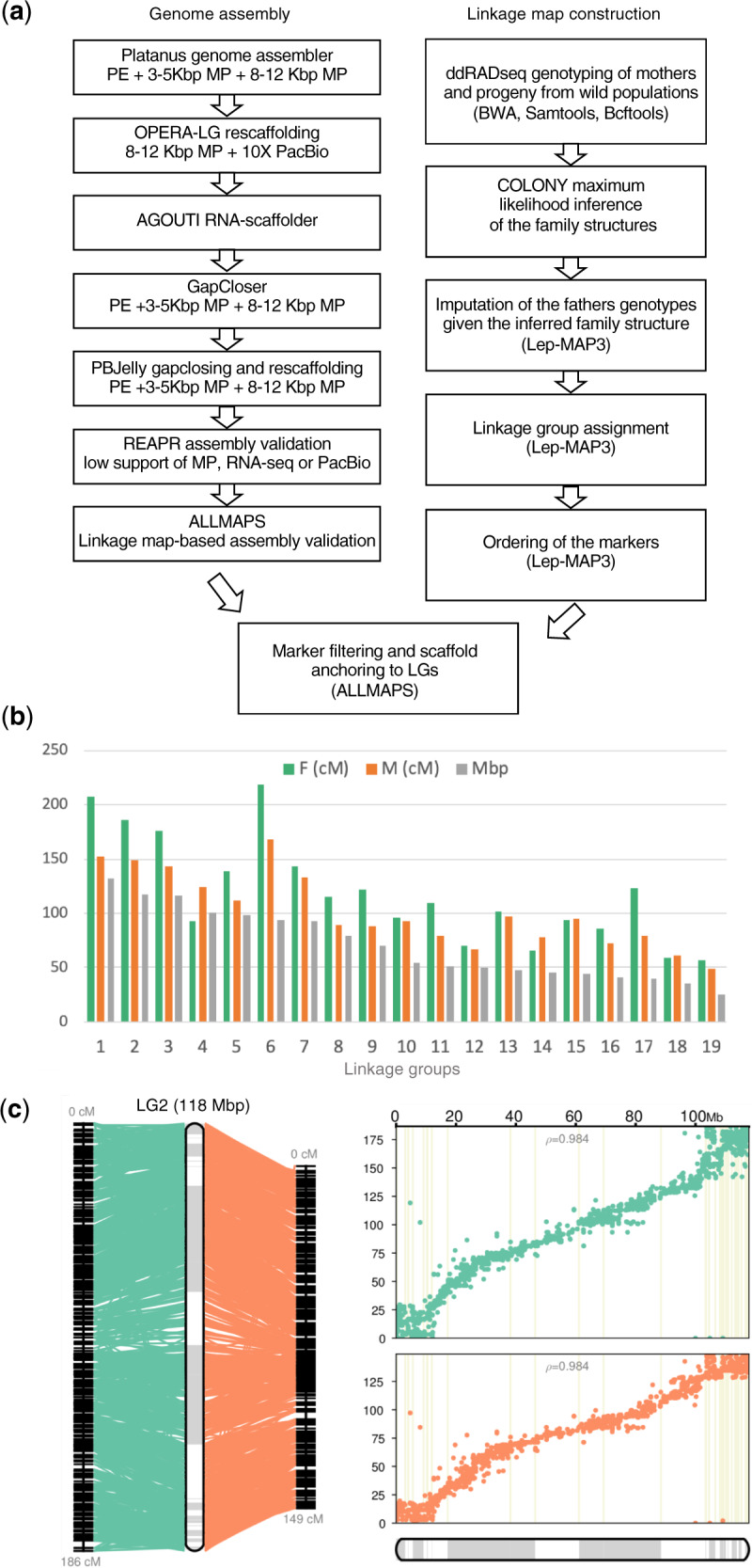
The genome assembly and linkage map construction for the common lizard, *Zootoca vivipara.* (*a*) The genome assembly and linkage map generation pipelines used in the study. (*b*) The length of the male (M), female (F), and consensus linkage groups. (*c*) An example of Linkage Group 2 based on the male (orange) and female (green) genetic maps. Pearson correlation coefficients between the physical (*X* axis) and genetic (*Y* axis) distances are indicated. Gray and white bars represent scaffolds.

At the final stage of the assembly, we closed gaps using the GapCloser v1.12 module in SoapDenovo2 (Luo et al. 2012) with all the available Illumina reads and then with the PBJelly v15.8.24 (English et al. 2012) long-read based algorithm. PacBio reads were error-corrected using Canu v1.5 (Koren et al. 2017) prior to the assembly. The overall assembly process is depicted in figure 1*a*.

### Linkage Map Construction

In total, 205 individuals from 20 families of known mothers and progeny but without paternal data were sampled nonlethally from the Gailtal region in Austria (permission from Bezirkshauptmannschaft Hermagor HE3-NS-959/2013). Individuals were sampled from the Central Viviparous II and Eastern Oviparous lineages, at a site where some admixture occurs (McLennan et al. 2019). DNA was extracted from tail clip tissue using the Dneasy Blood and Tissue Kit. Genomic libraries were constructed using double-digest restriction-site associated DNA sequencing following methods in Recknagel et al. (2018). Libraries were sequenced at Edinburgh Genomics on two lanes of Illumina HiSeq4000 with 150-bp PE reads. Reads were aligned to the genomic scaffolds and SNPs were called using bcftools (Li 2011) with successive family assignment (see supplementary text, Supplementary Material online).

Next, we used Lep-MAP3 v0.2 (Rastas 2017) to convert VCF files and produce the male and female linkage maps. At the final stage of linkage map construction, we arranged scaffolds into linkage groups with ALLMAPS (Tang et al. 2015), using both the male and female linkage maps simultaneously (supplementary text, Supplementary Material online).

### Assembly QC and Validation

We used REAPR v1.0.18 (Hunt et al. 2013) with mate-pairs and fragment coverage of PacBio and RNA-seq reads to validate the assembly. Finally, the assembly was validated and integrated with the linkage map (supplementary text, Supplementary Material online).

### Genome Annotation

To annotate we employed homology-based (GeMoMa 1.4.2; Keilwagen et al. 2016), ab initio prediction (AUGUSTUS v3.3; Stanke et al. 2006), and RNA-seq-based methods (StringTie v1.3.1c; Pertea et al. 2015), which were combined using EVidenceModeler v1.1.1 (Haas et al. 2008) during two stages. First, the consensus gene-models were calculated and extracted. Then, the consensus proteins were blasted against the Swiss-Prot (Boutet et al. 2007) database using DIAMOND v0.9.13 (Buchfink et al. 2015) and genes without any homology to the database were excluded (supplementary text, Supplementary Material online).

### Comparative Analysis

We identified single-copy orthologues from 16 published squamate genomes using Orthofinder (Emms and Kelly 2015). A phylogenetic tree of the aligned protein sequences was constructed in RAxML v.8.2.9 (Stamatakis 2014). Whole-genome alignment of the *Z. vivipara* assembly was performed against masked *Podarcis muralis* and *Crotalus viridis* genome assemblies using LASTZ (Harris 2007) (supplementary text, Supplementary Material online).

## Results and Discussion

### Genome Assembly

After read filtering and correction, we received 343M shotgun PE reads, 78M reads from 3- to 5-kb mate-pair libraries, and 53M reads from 8- to 12-kb mate-pair libraries from the short-read Illumina sequencing data (supplementary table S1, Supplementary Material online). These data were used to build contigs and scaffolds with the Platanus assembler along with 102M PE and 164M SE reads that were a by-product after trimming and filtering the PE and mate-pairs (only for contig assembly, supplementary table S1, Supplementary Material online).

The first scaffolds produced with Platanus had N50 metrics equal to 5.35 Mb and consisted of 366.9k contigs with a N50 of 5.23 kb (supplementary table S2, Supplementary Material online). After rescaffolding the assembly with Opera-LG using PacBio data (1.7 million reads) and the 8–12 kb mate-pairs, we doubled the N50 scaffold length to 12.52 Mb. Subsequent rescaffolding with the RNA-seq information on splicing events and gap closing using short reads increased the N50 contig size to 83.4 kb. Finally, gap closing with long PacBio reads allowed us to additionally increase the contig length distribution size to achieve an N50 of 220.4 kb.

The REAPR pipeline customized with additional PacBio and RNA-seq data allowed us to identify 1,733 likely erroneous joins between contigs, mostly at the ends of scaffolds that were then further broken. In sum, the assembled scaffolds were high quality and highly contiguous, benefiting from the combination of data types.

### Linkage Map Construction and Scaffold Anchoring

We used linkage mapping as an established approach for chromosome-level assembly and which provides additional information on recombination rates, physical to genetic distances, and sex-specific recombination (Fierst 2015). Sequencing for linkage maps generated 643M clean PE reads that were used, representing 20 families of mothers and offspring. Through a stringent probabilistic estimation of family structure and parent assignment, we found widespread multiple paternities. Specifically, four families had a single father whereas all others had from two to four fathers, with mean of 3.7 progeny per half-sib family (supplementary table S5, Supplementary Material online). This agrees with other research suggesting multiple paternity is abundant in common lizards (Laloi et al. 2004; Fitze et al. 2005).

We retained 109,640 high-quality biallelic SNPs and used them for imputing the missing genotypes of fathers in a probabilistic framework. The high genomic diversity made imputation efficient due to a large number of highly polymorphic SNPs with heterozygous positions. At the first stage of linkage map construction, 17,210 markers were assigned to 19 linkage groups (from 395 to 1,648 markers per LG, LOD score = 10.7), in agreement with the *Z. vivipara* karyotype with 17 autosomes and the Z and W sex chromosomes (2*n* = 36 chromosomes including ZZ/Zw sex chromosomes) specific for these lineages (Kupriyanova et al. 2014). At the next step, an additional 7,177 markers were assigned to these LGs with a minimal LOD score of 9. Finally, we had 1.27 and 1.24 markers per cM for the male (1,929.24 cM) and female (2,263.13 cM) linkage maps with 2,487 and 2,845 unique points, respectively (supplementary table S3, Supplementary Material online). The relatively low rate (21%, 24,387/109,640) of linkage-informative, high-quality SNPs that were assigned to the final linkage map can partially be attributed to the imputation of father genotypes and our stringent criteria for inclusion (supplementary text, Supplementary Material online).

We anchored 91.2% and oriented 89.5% of the assembly using the linkage map (supplementary table S4, Supplementary Material online). The physical size of linkage groups varied from 24.9 to 131.77 Mb and physical positions of markers strongly correlated with the linkage-based positions on the map (fig. 1*b* and *c*). The average resolution of the male and female linkage maps was 0.67 and 0.59 Mb per cM, respectively (supplementary table S3, Supplementary Material online).

At the final stage we identified and broke 30 intrascaffold regions that showed signs of misassembly according to the linkage map data. After this validation step, the formal assembly quality metrics slightly reduced (scaffold N50 by 1.23–11.52 Mb), but still indicated a high level of assembly contiguity (supplementary table S2, Supplementary Material online). Therefore, given that these are all within the same species, a relatively stable autosomal karyotype was known (Odierna et al. 2001), very few scaffolds were reassembled by linkage map information, and the physical and genetic distances are concordant (fig. 1), the use of multiple lineages did not have significant consequences on the chromosome-level assembly. However, future lineage-specific assemblies would be valuable and informative.

To further quantify the quality of the final scaffolds, we estimated the number of recovered Tetrapoda single-copy orthologues (BUSCO) in the assembled genome. We found that 94% of orthologues were completely assembled (with 1.3% of them being duplicated), 3.7% were fragmented, and 2.3% of the 3,950 benchmarked genes were missed. This metric is comparable to other recently assembled high quality genomes (Andrade et al. 2019; Suryamohan et al. 2020) and indicates that the assembly was of high quality with only minor parts of the genome being fragmented.

### Genome Annotation

Homology-based GeMoMa allowed us to identify 21,187 high quality gene-models with strong homology to chicken, Japanese gecko, and anole lizard genomes. The ab initio AUGUSTUS pipeline identified 15,637 gene-models which were finally combined using EVidenceModeler with 28,473 RNA-seq-based Transdecoder and GeMoMa gene models. After filtering out genes without any detected homology to the Swiss-Prot database, we received a final set of 19,829 protein-coding gene models. This is slightly lower than the three other lacertid species sequenced (Kolora et al. 2018; Andrade et al. 2019) and other squamates (Eckalbar et al. 2013; Suryamohan et al. 2020) which used less stringent filtering criteria but comparable to NCBI annotated genomes (19,431 protein coding genes for *Anolis carolinensis*; 19,535 for *Gekko japonicus*; and 18,971 for *Pogona vitticeps*).

### Genome Size

The estimated genome size of *Z. vivipara* was ≈1.345 Gb based on SGA k-mer distribution analysis, agreeing with earlier flow-cytometry based reports (1.035–1.515 Gb) (Vinogradov 1998). The final assembly length, including all linkage groups and unanchored scaffolds, was 1.46 Gb.

### Comparative Analysis

**Fig. 2.**
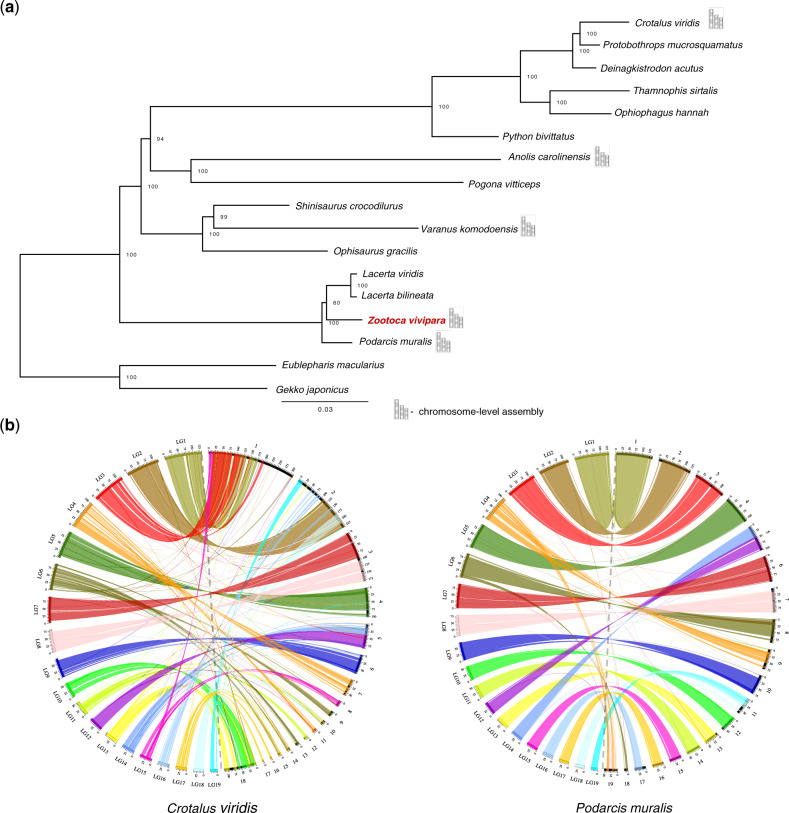
*Zootoca vivipara* genome assembly in the context of other Squamata genomes. (*a*) Maximum-likelihood tree based on the 269 single-copy orthologs from available Squamata genomes. (*b*) Synteny between *Z. vivipara* chromosome-level assembly and snake (*Crotalus viridis*) and closely related wall lizard genome (*Podarcis muralis*).

This *Z. vivipara* genome is one of six chromosome-level assemblies of the species-rich squamates to date. The hybrid assembly strategy we employed allowed us to achieve superior contig and scaffold size to Illumina-only squamate assemblies: 220.4 kb for contig and 11.54 Mb for scaffold N50 size in *Z. vivipara* versus *O. gracilis* genome with 42.8 kb contig and 1.27 Mb size as an example of one of the best Illumina-only squamate genome assemblies (Song et al. 2015). The contiguity of these *Z. vivipara* contigs is comparable to the other hybrid assembly-based genomes (Kolora et al. 2018) but is less than assemblies mainly generated by long-reads (Andrade et al. 2019). Our maximum-likelihood analysis resolved a phylogeny with *Z. vivipara* in a clade together with the recently assembled wall lizard (*P. muralis)* and two other *Lacerta* species (fig. 2*a*). The lacertid clade was, as expected (Irisarri et al. 2017), deeply divergent from other groups. Whole-genome alignment between *Z. vivipara* and the <40 Ma divergent (Garcia-Porta et al. 2019) *P. muralis* genome (Andrade et al. 2019) demonstrated a high level of synteny. The divergence time between common lizards and the rattlesnake, *C. viridis*, is >160 Ma (Pyron and Burbrink 2014), but nonetheless synteny was broadly conserved (fig. 2*b*). However, synteny analyses also demonstrated dynamic genome rearrangements between these distant lineages and many inter and intrachromosomal changes. In summary, the *Z. vivipara* genome shows high levels of contiguity and synteny with other squamate species, decreasing in synteny with divergence time.

## Conclusions

Here, we report a chromosome-level genome assembly for the wide-ranging, cold-adapted and reproductively bimodal common lizard, *Z. vivipara*. The final assembly contains 19 linkage groups with almost 90% of the genome anchored and oriented, and assembly length is 1.46 Gb. We annotated 19,829 protein-coding genes and inferred high quality BUSCO metrics, with 97.7% of Tetrapoda-specific single-copy orthologues recovered (only 3.7% fragmented). We applied a novel linkage mapping approach from multiple families with absent paternal information and multiple paternity structure, which could be applied to other sexually reproducing systems in which one parent and sibs are known but the other parent must be imputed. This genome assembly will be a useful resource for a wide range of studies on the fascinating evolutionary diversity of squamate reptiles.

## Supplementary Material

Supplementary data are available at *Genome Biology and Evolution* online.

## Supplementary Material

evaa161_Supplementary_Data

## Notes

**Data deposition:** This project has been deposited at NCBI under the accessions PRJNA610958 (genome assembly and raw sequencing data) and PRJNA626507 (linkage map raw data), and Glasgow University Research Repository http://researchdata.gla.ac.uk/1005 (gene annotation).
