# Supplementary material for: Chromosome-Level Assembly of the Common Lizard (Zootoca vivipara) Genome
Source: Genome Biol Evol. 2020 Aug 24;12(11):1953–60. doi: 10.1093/gbe/evaa161 (PMC7643610; doi:10.1093/gbe/evaa161)
Supplement: evaa161_Supplementary_Data [file evaa161_supplementary_data.docx]

**Supplementary Material for:**

**Chromosome-level assembly of the common lizard (*Zootoca vivipara*) genome**

Andrey A. Yurchenko, Hans Recknagel & Kathryn R. Elmer^*^

Institute of Biodiversity, Animal Health and Comparative Medicine, College of Medical, Veterinary and Life Sciences, University of Glasgow, Glasgow, G12 8QQ, Scotland, UK

* Author for Correspondence: Kathryn Elmer; Institute of Biodiversity, Animal Health and Comparative Medicine, College of Medical, Veterinary and Life Sciences, University of Glasgow, Glasgow, G12 8QQ, Scotland, UK; kathryn.elmer@glasgow.ac.uk

**Supplementary Text**

*Linkage map generation and family assignment*

Genotyping protocols by ddRADseq followed the methods in Recknagel et al. (2018). Initially, 1 ug of DNA was digested using restriction enzymes PstI-HF and MspI and subsequently cleaned with the Enzyme Reaction Cleanup kit (Qiagen). The amount of DNA in each offspring individual was then normalized to the sample with the lowest concentration within a library (275 ng in both libraries) to minimize coverage variation. DNA input for mothers was three times that for offspring (750 ng) to ensure higher coverage and therefore high confidence for maternal genotypes. Illumina-specific P1 and P2 adapters were ligated to the sticky ends generated by the restriction enzymes. The ligated DNA fragments were then multiplexed and size-selected using a Pippin Prep (Sage Science) for a ‘tight’ range of 150 – 210 bp. Seven separate PCR reactions (for details see Recknagel et al. 2015) were performed per library and combined (Peterson et al. 2012). Following PCR purification, libraries were electrophoresed on a 1.25% agarose gel, visualised with SYBRSafe (Life Technologies), and bands cut out manually to remove any remaining adapter dimers and fragments outside the size range. Product was extracted from the matrix using a MinElute Gel Extraction Kit (Qiagen). DNA libraries were then quantified using a Qubit Fluorometer with the dsDNA BR Assay and quality and quantity assessed using a TapeStation or Bioanalyzer (Agilent Technologies).

After Illumina sequencing (see main manuscript), we assessed the quality of the raw reads using FASTQC software (Andrews 2015) and removed low-quality reads and the right-end 50 bp of overlapping and technical sequences with Trimmomatic v0.36 (CROP:100 SLIDINGWINDOW:4:19). The cleaned reads were demultiplexed using Stacks v1.46 (Catchen et al. 2011) pipeline with command *process_radtags* (-c -q -r --inline_inline --renz_1 pstI --renz_2 mspI -i gzfastq --len_limit 5).

We aligned the ddRADSeq reads to the *Z. vivipara* scaffolds with BWA mem (Li & Durbin 2009) and prepared the sorted BAM files with Samtools (Li et al. 2009). Following the alignment, we called SNPs in all samples simultaneously using the *samtools mpileup | bcftools call* pipeline. For the *samtools mpileup* command, we only used reads with mapping quality equal to at least 45 (-q 45) and a minimal base quality equal to 20 (-Q 20) along with other options suitable for the ddRADSeq data (--count-orphans --ignore-overlaps -I -P illumine). For the *bcftools call* command we used the -m flag, which invokes multi-sample calling algorithm.

It is known that females of many reptile species, including *Zootoca vivipara*, can produce progeny from different males in within a single clutch (Laloi et al. 2004; Uller & Olsson 2008) and therefore we expected some clutches in our sample to have multiple fathers. In order to address this issue during linkage map construction and imputation of father genotypes, we used COLONY v2.0.6.3 (Jones & Wang 2010) to assign offspring from each clutch (=family) to half-sib families if they were inferred to have different fathers. We split the raw VCF file for linkage map construction by families using vcftools v0.1.15 (Danecek et al. 2011) and applied the following filters: minimal quality of a variant=700 (--minQ 700), minimal quality of a genotype=20 (--minGQ 20), only biallelic SNPs (--min-alleles 2 --max-alleles 2), minimal distance between the SNPs to reduce linkage=50 kbp (--thin 50000), no missing genotypes and indels (--max-missing-count 0 --remove-indels). Then the VCF files of each family were converted to COLONY format and 1900 random loci were chosen and tested with the following parameters to infer the family relationship: inbreeding absent, diploid species, polygamy for males and females, weak sibship prior, unknown population frequency, one long run, full-likelihood method, very high precision. Based on these results, offspring of each mother were clustered by inferred father, resulting in 1-4 half-sib families per mother. Patterns of variation within and across families were explored with a PCA. The family structure inferred with COLONY is presented in the Supplementary table S5.

The VCF files derived from the genome mapping and half-sib family inference step were filtered allowing minimal phred-scaled variant quality=500 (--minQ 500), minimal genotype quality=10 (--minGQ 10), minor allele count per loci=8 (--mac 8), minimum and maximum alleles per loci=2 (--min-alleles 2 --max-alleles 2), maximum missing count=20 (--max-missing-count 20). We used relatively liberal thresholds for filtering because the rest of the linkage map pipeline was based on genotype-likelihood values instead of the exact genotypes and therefore automatically accounts for error-probabilities of genotype calling, making the following imputation and linkage map construction steps fully probabilistic.

*Linkage map construction*

Initially the filtered VCF file was converted to the Lep-MAP3 posterior probabilities format with the developer’s script *vcf2posterior.awk*. Then we added pedigree information from a custom Excel spreadsheet at the header of the Lep-MAP3 posterior probabilities file and posterior values of zero for the father genotypes, which were absent in our dataset. Next, we imputed the missing father genotypes with the command *ParentCall2* using flags halfSibs=1, familyLimit=0.5, removeNonInformative=1 and outputParentPosterior=1.

In order to construct the linkage map, we used different combinations of parameters to empirically optimise the number of linkage groups and markers in them based on the highest LOD score. Following the Lep-MAP3 pipeline, first we separated linkage groups with the module *SeparateChromosomes2* using LOD score=10.7 (lodLimit=10.7) and at least 150 markers per linkage group. Then we added additional markers to the generated linkage groups using module *JoinSingles2All* with the minimal LOD score=9 (lodLimit=9), minimal difference to assign a marker to one or another linkage group=5 (lodDifference=5), and sequential iterations to assign markers (iterate=1). Finally, the markers were ordered using *OrderMarkers2* module with Kosambi distances (useKosambi=1), separately for each sex (sexAveraged=0) and in the course of eight subsequent iterations (numMergeIterations=8).

*Validation and Quality Control of the genome assembly*

In order to validate the assembly and avoid retaining erroneously joined contigs, we used REAPR v1.0.18 (Hunt et al. 2013), which utilizes mate-pair reads mapped to the assembly to find extensive drops in the fragment coverage along the genome; such signals can be attributed to erroneous contig joinings. To do so we mapped the 8-12 kbp mate-pair library reads to the genome using SMALT mapper (Ponsting & Ning 2010) as recommended by the REAPR developers and then identified suspicious regions in the genome. Additionally, we mapped PacBio and RNA-seq reads to the assembly using BWA mem and estimated fragment coverage at those suspicious regions using BEDTools v2.26.0 (Quinlan 2014). If the PacBio or RNA-seq fragment coverage was less than 2x, we broke the scaffolds in the identified suspicious regions.

Second, we used the *Z. vivipara* high-density linkage map to break potential contig misjoins using ALLMAPS v0.7.7 (Tang et al. 2015). After linkage group construction we applied the ALLMAPS command *split* with at least three significant matches to different linkage groups (--chunk=3), then the coordinates of the potential splits were refined based on the location of gaps in the assembly (*gaps* and *refine* commands), and scaffolds were broken in those coordinates. Then the 500 bp long regions surrounding the RAD-seq markers were extracted from the scaffold assembly (bedtools *getfasta*) and remapped with *blastn* (-max_target_seqs 1 -outfmt 6 -evalue 1e-100) to the broken assembly in order to build the final linkage groups with the ALLMAPS *path* command.

During the course of the genome assembly we explored different assembly methods and pipelines and controlled quality of the output using BUSCO v2.0.1 (Simão et al. 2015; tetrapoda_odb9 database with 3950 single-copy orthologs) and QUAST v4.4 software (Gurevich et al. 2013) quality metrics. BUSCO searches for a set of reliable single-copy orthologues that occur with the highest probability in the given taxonomic group. QUAST estimates different metrics of the genome contiguity and completeness. Metrics were compared at each stage.

*Genome Annotation*

For the homology-based gene prediction we used GeMoMa 1.4.2 (Keilwagen et al. 2016), which exploits dynamic programming and intron position conservation supported by RNA-seq data to precisely predict genes in evolutionary related genomes. We aligned the cleaned RNA-seq reads to the *Z. vivipara* scaffolds using STAR v020201 (Dobin et al. 2013) with the following settings: --twopass Mode Basic --seedSearchStartLmax 25 and extracted intron positions using GeMoMa *CLI ERE* command. Then we downloaded genomes and annotations of Japanese gecko *Gekko japonicus* (Liu et al. 2015), chicken *Gallus gallus* (Hillier et al. 2004), and green anole lizard *Anolis carolinensis* (Alföldi et al. 2011) from the NCBI RefSeq database (Pruitt et al. 2007) and extracted exons for each species finally converting them into amino acid sequences using GeMoMa *CLI Extractor* command. The translated exons were aligned to *Z. vivipara* scaffolds using *tblastn* (Gerts et al. 2006) in parallel with assistance of the GNU PARALLEL v20161022 software (Tange 2011). Then, the gene models were identified using the *CLI GeMoMa* module with the maximum allowed intron length equal to 50 kbp, the maximum number of transcripts per gene equal to 1, and at least 2 split reads needed to infer an intron border position with RNA-seq data. The gene predictions for each species were further combined and filtered using the GeMoMa Annotation Filter (*CLI GAF*) with the following settings: relative score filter =2, common border filter=1, and other settings were set as default.

To invoke RNA-seq evidence for the gene prediction we used StringTie v1.3.1c (Pertea et al. 2015) to assemble transcripts based on the STAR-aligned BAM files and then filtered and improved them using TACO v0.7.3 (Niknafs et al. 2016) with the following settings: --filter-min-expr 0.1, --max-isoforms 1. After, the transcripts were extracted from the scaffolds using the *gtf_genome_to_cdna_fasta.pl* script of the TransDecoder v5.0.2 pipeline (https://github.com/TransDecoder/TransDecoder) and coding sequences were identified in them with the TransDecoder.LongOrfs module and then blasted against the Swiss-Prot database (Boutet et al. 2007) using the DIAMOND v0.9.13 software (Buchfink et al. 2014) with the following settings: -c 1 -k 1 -p 60 -e 1e-5 --more-sensitive. Finally, we retained at most one open reading frame (ORF) per transcript using the TransDecoder.Predict module and hints from the DIAMOND *blastp* output and combined them with the TACO output to extract protein-coding gene models with RNA-seq evidence and homology to existing proteins with the script *cdna_alignment_orf_to_genome_orf.pl*.

The *de novo* annotation part was accomplished with the AUGUSTUS v3.3 software (Stanke et al. 2006) using scaffolds with repeats masked by WindowMasker v2.7.0 (Morgulis et al. 2006) and empirically optimised Hidden Markov Model from the BUSCO output (--species).

*Comparative analysis*

We searched for single-copy orthologs in 16 Squamata genomes (Aird et al. 2017; Alföldi et al. 2011; Andrade et al. 2019; Castoe, Bronikowski, et al. 2011; Castoe, de Koning, et al. 2011; Gao et al. 2017; Georges et al. 2015; Kolora et al. 2019; Lind et al. 2019; Liu et al. 2015; Song et al. 2015; Vonk et al. 2013; Xiong et al. 2016; Yin et al. 2016) for which coding sequences (CDSs) were available using Orthofinder with translated coding sequences (Emms & Kelly 2015). The nucleotide sequences of single copy orthologs between all the species (n=269) were aligned using codon-based PRANK (Löytynoja & Goldman 2010) aligner *(+F -codon*) and the maximum-likelihood tree was constructed in RAxML v.8.2.9 (Stamatakis 2014) using GTRGAMMAI and 1000 bootstrap replicates. Whole genome alignment of the *Z. vivipara* assembly was performed against masked *Podarcis muralis* and *Crotalus viridis* genome assemblies using LASTZ software with the *–-chain* option (Harris 2007) and visualised in Circos (Krzywinski et al. 2009).

**Supplementary tables**

**Supplementary table S1.** Sequencing used for the assembly. The total data for genome assembly (excluding linkage maps) was 741 M reads of DNA sequence and 195 M reads of RNA sequence.

| **Data type** | **Before filtering, million reads** | **After filtering, million reads** |
| --- | --- | --- |
| Illumina shotgun sequencing, PE 150 bp | 394 | 343 |
| 3-5 kbp mate-pairs, PE 150 bp | 197 | 78 |
| 8-12 kbp mate-pairs, PE 150 bp | 140 | 53 |
| PacBio (N50 11498 kbp) | 2,1 | 1,7 |
| RNA-seq, PE 150 bp | 357 | 195 |
| RAD-seq, PE 150 bp for the linkage map | 763 | 643 |
| SE from mate-pairs and shotgun sequencing | - | 164 |
| PE from mate-pairs and shotgun sequencing | - | 102 |

**Supplementary table S2.** The main steps and associated statistics of the genome assembly

| **Assembly step** | **Total length, bp** | **Number of contigs** | **Number of scaffolds** | **N50, contigs, bp** | **N50, scaffolds, bp** | **N75, scaffolds, bp** | **L75, scaffolds** | **Ns, %** | **GC, %** |
| --- | --- | --- | --- | --- | --- | --- | --- | --- | --- |
| Platanus scaffolding | 1,385,247,011 | 366,946 | 39,318 | 5,280 | 5,352,728 | 2,077,179 | 176 | 9.32 | 43.62 |
| OPERA-LG re-scaffolding | 1,445,088,735 | 366,946 | 21,986 | 5,280 | 12,518,550 | 4,255,225 | 82 | 13.08 | 43.62 |
| AGOUTI RNA scaffolder | 1,445,717,041 | 367,113 | 22,044 | 5,278 | 12,518,550 | 4,255,225 | 81 | 13.09 | 43.63 |
| GapCloser | 1,458,329,183 | 57,299 | 22,044 | 83,477 | 12,634,033 | 4,295,180 | 81 | 5.17 | 43.90 |
| PBJelly gap-closing and scaffolding | 1,470,820,215 | 41,168 | 21,930 | 220,452 | 12,767,756 | 4,360,152 | 80 | 3.40 | 43.97 |
| REAPR assembly validation | 1,464,313,252 | 42,935 | 25,587 | 219,985 | 12,749,897 | 4,421,500 | 79 | 2.97 | 43.97 |
| Final assembly after linkage map-based validation | 1,464,236,262 | 43,066 | 25,617 | 219,893 | 11,524,759 | 3,854,798 | 91 | 2.96 | 43.97 |

**Supplementary table S3.** Statistics for the *Zootoca vivipara* linkage map and the anchored assembly

| **LG** | **Length, cM** | | **Length, Mbp** | **Mbp/cM** | | **Number of markers** | | **Number of SNP** | **Markers per cM** | |
| --- | --- | --- | --- | --- | --- | --- | --- | --- | --- | --- |
|  | **M** | **F** |  | **M** | **F** | **M** | **F** |  | **M** | **F** |
| LG1 | 152.19 | 207.11 | 131.77 | 0.87 | 0.64 | 202 | 278 | 2032 | 1.33 | 1.34 |
| LG2 | 149.21 | 186.4 | 117.83 | 0.79 | 0.63 | 201 | 244 | 2265 | 1.35 | 1.31 |
| LG3 | 143.72 | 175.6 | 116.37 | 0.81 | 0.66 | 191 | 222 | 1716 | 1.33 | 1.26 |
| LG4 | 124.42 | 92.49 | 100.63 | 0.81 | 1.09 | 155 | 100 | 1164 | 1.25 | 1.08 |
| LG5 | 111.41 | 139.05 | 98.02 | 0.88 | 0.7 | 158 | 177 | 1280 | 1.42 | 1.27 |
| LG6 | 168.21 | 218.62 | 94.2 | 0.56 | 0.43 | 201 | 265 | 2295 | 1.19 | 1.21 |
| LG7 | 133.27 | 143.17 | 92.81 | 0.7 | 0.65 | 180 | 188 | 1747 | 1.35 | 1.31 |
| LG8 | 89.21 | 115.56 | 79.63 | 0.89 | 0.69 | 126 | 152 | 1270 | 1.41 | 1.32 |
| LG9 | 87.75 | 122.42 | 69.68 | 0.79 | 0.57 | 101 | 153 | 1054 | 1.15 | 1.25 |
| LG10 | 92.68 | 96.26 | 54.41 | 0.59 | 0.57 | 118 | 125 | 1119 | 1.27 | 1.3 |
| LG11 | 79.11 | 109.29 | 51.06 | 0.65 | 0.47 | 112 | 143 | 1187 | 1.42 | 1.31 |
| LG12 | 66.52 | 70.68 | 49.59 | 0.75 | 0.7 | 79 | 80 | 797 | 1.19 | 1.13 |
| LG13 | 97.45 | 102.02 | 48.21 | 0.49 | 0.47 | 139 | 140 | 1235 | 1.43 | 1.37 |
| LG14 | 78.12 | 65.76 | 46.01 | 0.59 | 0.7 | 91 | 80 | 751 | 1.16 | 1.22 |
| LG15 | 94.75 | 94 | 44.54 | 0.47 | 0.47 | 129 | 124 | 1310 | 1.36 | 1.32 |
| LG16 | 72.62 | 85.55 | 40.7 | 0.56 | 0.48 | 96 | 104 | 852 | 1.32 | 1.22 |
| LG17 | 78.78 | 123.32 | 39.44 | 0.5 | 0.32 | 101 | 145 | 1183 | 1.28 | 1.18 |
| LG18 | 61.36 | 59.53 | 35.46 | 0.58 | 0.6 | 61 | 63 | 580 | 0.99 | 1.06 |
| LG19 | 48.46 | 56.3 | 24.9 | 0.51 | 0.44 | 46 | 62 | 550 | 0.95 | 1.1 |
| Total/mean | 1929.24 | 2263.13 | 1335.27 | 0.67 | 0.59 | 2487 | 2845 | 24387 | 1.27 | 1.24 |

**Supplementary table S4.** Linkage map summary and association with scaffolds

| Statistics | Anchored | Oriented | Unplaced |
| --- | --- | --- | --- |
| Number of markers | 24,205 | 23,781 | 126 |
| Number of markers per Mbp | 18.1 | 18.1 | 1 |
| Scaffolds | 420 | 295 | 23,235 |
| Scaffolds with 1 marker | 60 | 0 | 38 |
| Scaffolds with 2 markers | 41 | 31 | 14 |
| Scaffolds with ≥ 4 markers | 286 | 256 | 8 |
| Total bases | 1,335,231,344 (91.2%) | 1,310,909,853 (89.5%) | 129,018,870 (8.8%) |

**Supplementary table S5.** Family structure and the corresponding individuals used for the linkage map construction. Clutch family ID – families inferred as progeny of a single clutch in the field. Halfsib family ID – families inferred using genetic information with multiple paternity consideration. Imputed father - fathers inferred from genetic information. Sex 0, 1 and 2 correspond to unknown, male and female respectively.

| Clutch family ID | Halfsib family ID | Individual | Imputed Father | Mother | Sex |
| --- | --- | --- | --- | --- | --- |
| 1 | 1_2 | ELT04348 | 1_2 | ELT03727 | 0 |
| 1 | 1_2 | ELT03727 | 0 | 0 | 2 |
| 1 | 1_2 | 1_2 | 0 | 0 | 1 |
| 1 | 1_3 | ELT04349 | 1_3 | ELT03727 | 0 |
| 1 | 1_3 | ELT04350 | 1_3 | ELT03727 | 0 |
| 1 | 1_3 | ELT04352 | 1_3 | ELT03727 | 0 |
| 1 | 1_3 | ELT04353 | 1_3 | ELT03727 | 0 |
| 1 | 1_3 | ELT03727 | 0 | 0 | 2 |
| 1 | 1_3 | 1_3 | 0 | 0 | 1 |
| 1 | 1_4 | ELT04351 | 1_4 | ELT03727 | 0 |
| 1 | 1_4 | ELT04354 | 1_4 | ELT03727 | 0 |
| 1 | 1_4 | ELT04357 | 1_4 | ELT03727 | 0 |
| 1 | 1_4 | ELT03727 | 0 | 0 | 2 |
| 1 | 1_4 | 1_4 | 0 | 0 | 1 |
| 1 | 1_5 | ELT04355 | 1_5 | ELT03727 | 0 |
| 1 | 1_5 | ELT04356 | 1_5 | ELT03727 | 0 |
| 1 | 1_5 | ELT03727 | 0 | 0 | 2 |
| 1 | 1_5 | 1_5 | 0 | 0 | 1 |
| 2 | 2_2 | ELT04334 | 2_2 | ELT03762 | 0 |
| 2 | 2_2 | ELT04335 | 2_2 | ELT03762 | 0 |
| 2 | 2_2 | ELT04336 | 2_2 | ELT03762 | 0 |
| 2 | 2_2 | ELT04338 | 2_2 | ELT03762 | 0 |
| 2 | 2_2 | ELT04340 | 2_2 | ELT03762 | 0 |
| 2 | 2_2 | ELT03762 | 0 | 0 | 2 |
| 2 | 2_2 | 2_2 | 0 | 0 | 1 |
| 2 | 2_3 | ELT04337 | 2_3 | ELT03762 | 0 |
| 2 | 2_3 | ELT04339 | 2_3 | ELT03762 | 0 |
| 2 | 2_3 | ELT04341 | 2_3 | ELT03762 | 0 |
| 2 | 2_3 | ELT04342 | 2_3 | ELT03762 | 0 |
| 2 | 2_3 | ELT03762 | 0 | 0 | 2 |
| 2 | 2_3 | 2_3 | 0 | 0 | 1 |
| 3 | 3_2 | ELT04319 | 3_2 | ELT03765 | 0 |
| 3 | 3_2 | ELT04320 | 3_2 | ELT03765 | 0 |
| 3 | 3_2 | ELT04321 | 3_2 | ELT03765 | 0 |
| 3 | 3_2 | ELT04322 | 3_2 | ELT03765 | 0 |
| 3 | 3_2 | ELT04323 | 3_2 | ELT03765 | 0 |
| 3 | 3_2 | ELT04324 | 3_2 | ELT03765 | 0 |
| 3 | 3_2 | ELT04325 | 3_2 | ELT03765 | 0 |
| 3 | 3_2 | ELT04326 | 3_2 | ELT03765 | 0 |
| 3 | 3_2 | ELT04327 | 3_2 | ELT03765 | 0 |
| 3 | 3_2 | ELT03765 | 0 | 0 | 2 |
| 3 | 3_2 | 3_2 | 0 | 0 | 1 |
| 4 | 4_2 | ELT04284 | 4_2 | ELT03865 | 0 |
| 4 | 4_2 | ELT04292 | 4_2 | ELT03865 | 0 |
| 4 | 4_2 | ELT03865 | 0 | 0 | 2 |
| 4 | 4_2 | 4_2 | 0 | 0 | 1 |
| 4 | 4_3 | ELT04285 | 4_3 | ELT03865 | 0 |
| 4 | 4_3 | ELT04286 | 4_3 | ELT03865 | 0 |
| 4 | 4_3 | ELT04287 | 4_3 | ELT03865 | 0 |
| 4 | 4_3 | ELT04288 | 4_3 | ELT03865 | 0 |
| 4 | 4_3 | ELT04289 | 4_3 | ELT03865 | 0 |
| 4 | 4_3 | ELT04290 | 4_3 | ELT03865 | 0 |
| 4 | 4_3 | ELT04291 | 4_3 | ELT03865 | 0 |
| 4 | 4_3 | ELT03865 | 0 | 0 | 2 |
| 4 | 4_3 | 4_3 | 0 | 0 | 1 |
| 5 | 5_2 | ELT04188 | 5_2 | ELT03912 | 0 |
| 5 | 5_2 | ELT04189 | 5_2 | ELT03912 | 0 |
| 5 | 5_2 | ELT04190 | 5_2 | ELT03912 | 0 |
| 5 | 5_2 | ELT04191 | 5_2 | ELT03912 | 0 |
| 5 | 5_2 | ELT04192 | 5_2 | ELT03912 | 0 |
| 5 | 5_2 | ELT04193 | 5_2 | ELT03912 | 0 |
| 5 | 5_2 | ELT04194 | 5_2 | ELT03912 | 0 |
| 5 | 5_2 | ELT04195 | 5_2 | ELT03912 | 0 |
| 5 | 5_2 | ELT04196 | 5_2 | ELT03912 | 0 |
| 5 | 5_2 | ELT04197 | 5_2 | ELT03912 | 0 |
| 5 | 5_2 | ELT04198 | 5_2 | ELT03912 | 0 |
| 5 | 5_2 | ELT04199 | 5_2 | ELT03912 | 0 |
| 5 | 5_2 | ELT03912 | 0 | 0 | 2 |
| 5 | 5_2 | 5_2 | 0 | 0 | 1 |
| 6 | 6_2 | ELT03945 | 6_2 | ELT03916 | 0 |
| 6 | 6_2 | ELT03946 | 6_2 | ELT03916 | 0 |
| 6 | 6_2 | ELT03947 | 6_2 | ELT03916 | 0 |
| 6 | 6_2 | ELT03948 | 6_2 | ELT03916 | 0 |
| 6 | 6_2 | ELT03949 | 6_2 | ELT03916 | 0 |
| 6 | 6_2 | ELT03950 | 6_2 | ELT03916 | 0 |
| 6 | 6_2 | ELT03951 | 6_2 | ELT03916 | 0 |
| 6 | 6_2 | ELT03952 | 6_2 | ELT03916 | 0 |
| 6 | 6_2 | ELT03953 | 6_2 | ELT03916 | 0 |
| 6 | 6_2 | ELT03916 | 0 | 0 | 2 |
| 6 | 6_2 | 6_2 | 0 | 0 | 1 |
| 7 | 7_2 | ELT05723 | 7_2 | ELT04819 | 0 |
| 7 | 7_2 | ELT05726 | 7_2 | ELT04819 | 0 |
| 7 | 7_2 | ELT05728 | 7_2 | ELT04819 | 0 |
| 7 | 7_2 | ELT05729 | 7_2 | ELT04819 | 0 |
| 7 | 7_2 | ELT05730 | 7_2 | ELT04819 | 0 |
| 7 | 7_2 | ELT04819 | 0 | 0 | 2 |
| 7 | 7_2 | 7_2 | 0 | 0 | 1 |
| 7 | 7_3 | ELT05724 | 7_3 | ELT04819 | 0 |
| 7 | 7_3 | ELT05725 | 7_3 | ELT04819 | 0 |
| 7 | 7_3 | ELT05727 | 7_3 | ELT04819 | 0 |
| 7 | 7_3 | ELT05731 | 7_3 | ELT04819 | 0 |
| 7 | 7_3 | ELT04819 | 0 | 0 | 2 |
| 7 | 7_3 | 7_3 | 0 | 0 | 1 |
| 8 | 8_2 | ELT06239 | 8_2 | ELT05028 | 0 |
| 8 | 8_2 | ELT06240 | 8_2 | ELT05028 | 0 |
| 8 | 8_2 | ELT06241 | 8_2 | ELT05028 | 0 |
| 8 | 8_2 | ELT06242 | 8_2 | ELT05028 | 0 |
| 8 | 8_2 | ELT06243 | 8_2 | ELT05028 | 0 |
| 8 | 8_2 | ELT06244 | 8_2 | ELT05028 | 0 |
| 8 | 8_2 | ELT06245 | 8_2 | ELT05028 | 0 |
| 8 | 8_2 | ELT06248 | 8_2 | ELT05028 | 0 |
| 8 | 8_2 | ELT05028 | 0 | 0 | 2 |
| 8 | 8_2 | 8_2 | 0 | 0 | 1 |
| 8 | 8_3 | ELT06246 | 8_3 | ELT05028 | 0 |
| 8 | 8_3 | ELT06247 | 8_3 | ELT05028 | 0 |
| 8 | 8_3 | ELT05028 | 0 | 0 | 2 |
| 8 | 8_3 | 8_3 | 0 | 0 | 1 |
| 9 | 9_2 | ELT05817 | 9_2 | ELT04868 | 0 |
| 9 | 9_2 | ELT05821 | 9_2 | ELT04868 | 0 |
| 9 | 9_2 | ELT05822 | 9_2 | ELT04868 | 0 |
| 9 | 9_2 | ELT05824 | 9_2 | ELT04868 | 0 |
| 9 | 9_2 | ELT04868 | 0 | 0 | 2 |
| 9 | 9_2 | 9_2 | 0 | 0 | 1 |
| 9 | 9_4 | ELT05819 | 9_4 | ELT04868 | 0 |
| 9 | 9_4 | ELT05820 | 9_4 | ELT04868 | 0 |
| 9 | 9_4 | ELT05823 | 9_4 | ELT04868 | 0 |
| 9 | 9_4 | ELT04868 | 0 | 0 | 2 |
| 9 | 9_4 | 9_4 | 0 | 0 | 1 |
| 10 | 10_2 | ELT06035 | 10_2 | ELT04946 | 0 |
| 10 | 10_2 | ELT06036 | 10_2 | ELT04946 | 0 |
| 10 | 10_2 | ELT06038 | 10_2 | ELT04946 | 0 |
| 10 | 10_2 | ELT06041 | 10_2 | ELT04946 | 0 |
| 10 | 10_2 | ELT04946 | 0 | 0 | 2 |
| 10 | 10_2 | 10_2 | 0 | 0 | 1 |
| 10 | 10_3 | ELT06037 | 10_3 | ELT04946 | 0 |
| 10 | 10_3 | ELT06042 | 10_3 | ELT04946 | 0 |
| 10 | 10_3 | ELT04946 | 0 | 0 | 2 |
| 10 | 10_3 | 10_3 | 0 | 0 | 1 |
| 10 | 10_4 | ELT06039 | 10_4 | ELT04946 | 0 |
| 10 | 10_4 | ELT06040 | 10_4 | ELT04946 | 0 |
| 10 | 10_4 | ELT04946 | 0 | 0 | 2 |
| 10 | 10_4 | 10_4 | 0 | 0 | 1 |
| 10 | 10_5 | ELT06043 | 10_5 | ELT04946 | 0 |
| 10 | 10_5 | ELT04946 | 0 | 0 | 2 |
| 10 | 10_5 | 10_5 | 0 | 0 | 1 |
| 12 | 12_2 | ELT06097 | 12_2 | ELT04968 | 0 |
| 12 | 12_2 | ELT04968 | 0 | 0 | 2 |
| 12 | 12_2 | 12_2 | 0 | 0 | 1 |
| 12 | 12_3 | ELT06098 | 12_3 | ELT04968 | 0 |
| 12 | 12_3 | ELT04968 | 0 | 0 | 2 |
| 12 | 12_3 | 12_3 | 0 | 0 | 1 |
| 12 | 12_4 | ELT06099 | 12_4 | ELT04968 | 0 |
| 12 | 12_4 | ELT06100 | 12_4 | ELT04968 | 0 |
| 12 | 12_4 | ELT06101 | 12_4 | ELT04968 | 0 |
| 12 | 12_4 | ELT06104 | 12_4 | ELT04968 | 0 |
| 12 | 12_4 | ELT04968 | 0 | 0 | 2 |
| 12 | 12_4 | 12_4 | 0 | 0 | 1 |
| 12 | 12_5 | ELT06102 | 12_5 | ELT04968 | 0 |
| 12 | 12_5 | ELT06103 | 12_5 | ELT04968 | 0 |
| 12 | 12_5 | ELT04968 | 0 | 0 | 2 |
| 12 | 12_5 | 12_5 | 0 | 0 | 1 |
| 13 | 13_2 | ELT06370 | 13_2 | ELT05108 | 0 |
| 13 | 13_2 | ELT06375 | 13_2 | ELT05108 | 0 |
| 13 | 13_2 | ELT05108 | 0 | 0 | 2 |
| 13 | 13_2 | 13_2 | 0 | 0 | 1 |
| 13 | 13_3 | ELT06371 | 13_3 | ELT05108 | 0 |
| 13 | 13_3 | ELT06373 | 13_3 | ELT05108 | 0 |
| 13 | 13_3 | ELT06376 | 13_3 | ELT05108 | 0 |
| 13 | 13_3 | ELT06377 | 13_3 | ELT05108 | 0 |
| 13 | 13_3 | ELT05108 | 0 | 0 | 2 |
| 13 | 13_3 | 13_3 | 0 | 0 | 1 |
| 13 | 13_4 | ELT06372 | 13_4 | ELT05108 | 0 |
| 13 | 13_4 | ELT05108 | 0 | 0 | 2 |
| 13 | 13_4 | 13_4 | 0 | 0 | 1 |
| 13 | 13_5 | ELT06374 | 13_5 | ELT05108 | 0 |
| 13 | 13_5 | ELT05108 | 0 | 0 | 2 |
| 13 | 13_5 | 13_5 | 0 | 0 | 1 |
| 14 | 14_2 | ELT07639 | 14_2 | ELT07064 | 0 |
| 14 | 14_2 | ELT07640 | 14_2 | ELT07064 | 0 |
| 14 | 14_2 | ELT07064 | 0 | 0 | 2 |
| 14 | 14_2 | 14_2 | 0 | 0 | 1 |
| 14 | 14_3 | ELT07641 | 14_3 | ELT07064 | 0 |
| 14 | 14_3 | ELT07642 | 14_3 | ELT07064 | 0 |
| 14 | 14_3 | ELT07643 | 14_3 | ELT07064 | 0 |
| 14 | 14_3 | ELT07644 | 14_3 | ELT07064 | 0 |
| 14 | 14_3 | ELT07064 | 0 | 0 | 2 |
| 14 | 14_3 | 14_3 | 0 | 0 | 1 |
| 14 | 14_4 | ELT07645 | 14_4 | ELT07064 | 0 |
| 14 | 14_4 | ELT07646 | 14_4 | ELT07064 | 0 |
| 14 | 14_4 | ELT07647 | 14_4 | ELT07064 | 0 |
| 14 | 14_4 | ELT07648 | 14_4 | ELT07064 | 0 |
| 14 | 14_4 | ELT07064 | 0 | 0 | 2 |
| 14 | 14_4 | 14_4 | 0 | 0 | 1 |
| 15 | 15_2 | ELT07532 | 15_2 | ELT07074 | 0 |
| 15 | 15_2 | ELT07533 | 15_2 | ELT07074 | 0 |
| 15 | 15_2 | ELT07534 | 15_2 | ELT07074 | 0 |
| 15 | 15_2 | ELT07535 | 15_2 | ELT07074 | 0 |
| 15 | 15_2 | ELT07536 | 15_2 | ELT07074 | 0 |
| 15 | 15_2 | ELT07537 | 15_2 | ELT07074 | 0 |
| 15 | 15_2 | ELT07538 | 15_2 | ELT07074 | 0 |
| 15 | 15_2 | ELT07539 | 15_2 | ELT07074 | 0 |
| 15 | 15_2 | ELT07540 | 15_2 | ELT07074 | 0 |
| 15 | 15_2 | ELT07074 | 0 | 0 | 2 |
| 15 | 15_2 | 15_2 | 0 | 0 | 1 |
| 16 | 16_2 | ELT07975 | 16_2 | ELT07138 | 0 |
| 16 | 16_2 | ELT07981 | 16_2 | ELT07138 | 0 |
| 16 | 16_2 | ELT07982 | 16_2 | ELT07138 | 0 |
| 16 | 16_2 | ELT07138 | 0 | 0 | 2 |
| 16 | 16_2 | 16_2 | 0 | 0 | 1 |
| 16 | 16_3 | ELT07976 | 16_3 | ELT07138 | 0 |
| 16 | 16_3 | ELT07977 | 16_3 | ELT07138 | 0 |
| 16 | 16_3 | ELT07978 | 16_3 | ELT07138 | 0 |
| 16 | 16_3 | ELT07979 | 16_3 | ELT07138 | 0 |
| 16 | 16_3 | ELT07980 | 16_3 | ELT07138 | 0 |
| 16 | 16_3 | ELT07138 | 0 | 0 | 2 |
| 16 | 16_3 | 16_3 | 0 | 0 | 1 |
| 16 | 16_4 | ELT07984 | 16_4 | ELT07138 | 0 |
| 16 | 16_4 | ELT07986 | 16_4 | ELT07138 | 0 |
| 16 | 16_4 | ELT07138 | 0 | 0 | 2 |
| 16 | 16_4 | 16_4 | 0 | 0 | 1 |
| 16 | 16_5 | ELT07985 | 16_5 | ELT07138 | 0 |
| 16 | 16_5 | ELT07138 | 0 | 0 | 2 |
| 16 | 16_5 | 16_5 | 0 | 0 | 1 |
| 17 | 17_2 | ELT07550 | 17_2 | ELT07274 | 0 |
| 17 | 17_2 | ELT07551 | 17_2 | ELT07274 | 0 |
| 17 | 17_2 | ELT07552 | 17_2 | ELT07274 | 0 |
| 17 | 17_2 | ELT07553 | 17_2 | ELT07274 | 0 |
| 17 | 17_2 | ELT07554 | 17_2 | ELT07274 | 0 |
| 17 | 17_2 | ELT07555 | 17_2 | ELT07274 | 0 |
| 17 | 17_2 | ELT07556 | 17_2 | ELT07274 | 0 |
| 17 | 17_2 | ELT07274 | 0 | 0 | 2 |
| 17 | 17_2 | 17_2 | 0 | 0 | 1 |
| 17 | 17_3 | ELT07557 | 17_3 | ELT07274 | 0 |
| 17 | 17_3 | ELT07274 | 0 | 0 | 2 |
| 17 | 17_3 | 17_3 | 0 | 0 | 1 |
| 18 | 18_2 | ELT07511 | 18_2 | ELT07327 | 0 |
| 18 | 18_2 | ELT07512 | 18_2 | ELT07327 | 0 |
| 18 | 18_2 | ELT07513 | 18_2 | ELT07327 | 0 |
| 18 | 18_2 | ELT07515 | 18_2 | ELT07327 | 0 |
| 18 | 18_2 | ELT07516 | 18_2 | ELT07327 | 0 |
| 18 | 18_2 | ELT07517 | 18_2 | ELT07327 | 0 |
| 18 | 18_2 | ELT07327 | 0 | 0 | 2 |
| 18 | 18_2 | 18_2 | 0 | 0 | 1 |
| 18 | 18_3 | ELT07514 | 18_3 | ELT07327 | 0 |
| 18 | 18_3 | ELT07518 | 18_3 | ELT07327 | 0 |
| 18 | 18_3 | ELT07519 | 18_3 | ELT07327 | 0 |
| 18 | 18_3 | ELT07327 | 0 | 0 | 2 |
| 18 | 18_3 | 18_3 | 0 | 0 | 1 |
| 19 | 19_2 | ELT08053 | 19_2 | ELT07046 | 0 |
| 19 | 19_2 | ELT08055 | 19_2 | ELT07046 | 0 |
| 19 | 19_2 | ELT08056 | 19_2 | ELT07046 | 0 |
| 19 | 19_2 | ELT08057 | 19_2 | ELT07046 | 0 |
| 19 | 19_2 | ELT08058 | 19_2 | ELT07046 | 0 |
| 19 | 19_2 | ELT08059 | 19_2 | ELT07046 | 0 |
| 19 | 19_2 | ELT08060 | 19_2 | ELT07046 | 0 |
| 19 | 19_2 | ELT08061 | 19_2 | ELT07046 | 0 |
| 19 | 19_2 | ELT08062 | 19_2 | ELT07046 | 0 |
| 19 | 19_2 | ELT07046 | 0 | 0 | 2 |
| 19 | 19_2 | 19_2 | 0 | 0 | 1 |
| 19 | 19_3 | ELT08054 | 19_3 | ELT07046 | 0 |
| 19 | 19_3 | ELT07046 | 0 | 0 | 2 |
| 19 | 19_3 | 19_3 | 0 | 0 | 1 |
| 20 | 20_2 | ELT07791 | 20_2 | ELT07050 | 0 |
| 20 | 20_2 | ELT07792 | 20_2 | ELT07050 | 0 |
| 20 | 20_2 | ELT07796 | 20_2 | ELT07050 | 0 |
| 20 | 20_2 | ELT07797 | 20_2 | ELT07050 | 0 |
| 20 | 20_2 | ELT07799 | 20_2 | ELT07050 | 0 |
| 20 | 20_2 | ELT07050 | 0 | 0 | 2 |
| 20 | 20_2 | 20_2 | 0 | 0 | 1 |
| 20 | 20_3 | ELT07793 | 20_3 | ELT07050 | 0 |
| 20 | 20_3 | ELT07794 | 20_3 | ELT07050 | 0 |
| 20 | 20_3 | ELT07795 | 20_3 | ELT07050 | 0 |
| 20 | 20_3 | ELT07798 | 20_3 | ELT07050 | 0 |
| 20 | 20_3 | ELT07050 | 0 | 0 | 2 |
| 20 | 20_3 | 20_3 | 0 | 0 | 1 |
| 21 | 21_1 | 075xx1 | 21_1 | ELT07090 | 0 |
| 21 | 21_1 | ELT07523 | 21_1 | ELT07090 | 0 |
| 21 | 21_1 | ELT07524 | 21_1 | ELT07090 | 0 |
| 21 | 21_1 | ELT07525 | 21_1 | ELT07090 | 0 |
| 21 | 21_1 | ELT07526 | 21_1 | ELT07090 | 0 |
| 21 | 21_1 | ELT07528 | 21_1 | ELT07090 | 0 |
| 21 | 21_1 | ELT07529 | 21_1 | ELT07090 | 0 |
| 21 | 21_1 | ELT07531 | 21_1 | ELT07090 | 0 |
| 21 | 21_1 | ELT07090 | 0 | 0 | 2 |
| 21 | 21_1 | 21_1 | 0 | 0 | 1 |
| 21 | 21_2 | 075xx2 | 21_2 | ELT07090 | 0 |
| 21 | 21_2 | ELT07527 | 21_2 | ELT07090 | 0 |
| 21 | 21_2 | ELT07090 | 0 | 0 | 2 |
| 21 | 21_2 | 21_2 | 0 | 0 | 1 |

**Literature Cited**

Aird SD et al. 2017. Population genomic analysis of a pitviper reveals microevolutionary forces underlying venom chemistry. Genome Biol. Evol. 9:2640–2649. doi: 10.1093/gbe/evx199.

Alföldi J et al. 2011. The genome of the green anole lizard and a comparative analysis with birds and mammals. Nature. 477:587–591. doi: 10.1038/nature10390.

Andrade P et al. 2019. Regulatory changes in pterin and carotenoid genes underlie balanced color polymorphisms in the wall lizard. Proc. Natl. Acad. Sci. U. S. A. 116:5633–5642. doi: 10.1073/pnas.1820320116.

Andrews S. 2015. FASTQC A Quality Control tool for High Throughput Sequence Data. Babraham Inst. doi: http://www.bioinformatics.babraham.ac.uk/projects/fastqc.

Boutet E, Lieberherr D, Tognolli M, Schneider M, Bairoch A. 2007. UniProtKB/Swiss-Prot. Plant Bioinforma. 89–112. doi: 10.1007/978-1-59745-535-0_4.

Buchfink B, Xie C, Huson DH. 2014. Fast and sensitive protein alignment using DIAMOND. Nat. Methods. 12:59–60. doi: 10.1038/nmeth.3176.

Castoe TA, Bronikowski AM, et al. 2011. A proposal to sequence the genome of a garter snake (*Thamnophis sirtalis*). Stand. Genomic Sci. 4:257. doi: 10.4056/sigs.1664145.

Castoe TA, de Koning JAP, et al. 2011. Sequencing the genome of the Burmese python (*Python molurus bivittatus*) as a model for studying extreme adaptations in snakes. Genome Biol. 12:406. doi: 10.1186/gb-2011-12-7-406.

Catchen JM, Amores A, Hohenlohe P, Cresko W, Postlethwait JH. 2011. Stacks: Building and genotyping loci de novo from short-read sequences. G3 Genes Genom. Genet. 1:171–182. doi: 10.1534/g3.111.000240.

Danecek P et al. 2011. The variant call format and VCFtools. Bioinfomatics. 27:2156–2158. doi: 10.1093/bioinformatics/btr330.

Dobin A et al. 2013. STAR: Ultrafast universal RNA-seq aligner. Bioinfomatics. 29:15–21. doi: 10.1093/bioinformatics/bts635.

Emms DM, Kelly S. 2015. OrthoFinder: solving fundamental biases in whole genome comparisons dramatically improves orthogroup inference accuracy. Genome Biol. 16:157. doi: 10.1186/s13059-015-0721-2.

Gao J et al. 2017. Sequencing, de novo assembling, and annotating the genome of the endangered Chinese crocodile lizard *Shinisaurus crocodilurus*. Gigascience. 6:gix041. doi: 10.1093/gigascience/gix041.

Georges A et al. 2015. High-coverage sequencing and annotated assembly of the genome of the Australian dragon lizard *Pogona vitticeps*. Gigascience. 4:s13742-15. doi: 10.1186/s13742-015-0085-2.

Gerts EM, Yu YK, Agarwala R, Schäffer AA, Altschul SF. 2006. Composition-based statistics and translated nucleotide searches: Improving the TBLASTN module of BLAST. BMC Biol. 4:41. doi: 10.1186/1741-7007-4-41.

Gurevich A, Saveliev V, Vyahhi N, Tesler G. 2013. QUAST: Quality assessment tool for genome assemblies. Bioinfomatics. 29:1072–1075. doi: 10.1093/bioinformatics/btt086.

Harris RS. 2007. Improved Pairwise Alignment of Genomic DNA. PhD thesis, Pennsylvania State University. doi: 10.1016/j.brainres.2008.03.070.

Hillier LW et al. 2004. Sequence and comparative analysis of the chicken genome provide unique perspectives on vertebrate evolution. Nature. 423:695–777. doi: 10.1038/nature03154.

Hunt M et al. 2013. REAPR: A universal tool for genome assembly evaluation. Genome Biol. 14:R47. doi: 10.1186/gb-2013-14-5-r47.

Jones OR, Wang J. 2010. COLONY: A program for parentage and sibship inference from multilocus genotype data. Mol. Ecol. Resour. 10:551–555. doi: 10.1111/j.1755-0998.2009.02787.x.

Keilwagen J et al. 2016. Using intron position conservation for homology-based gene prediction. Nucleic Acids Res. 44:e89–e89. doi: 10.1093/nar/gkw092.

Kolora SRR et al. 2019. Divergent evolution in the genomes of closely related lacertids, *Lacerta viridis* and *L. bilineata*, and implications for speciation. Gigascience. 8:1–15. doi: 10.1093/gigascience/giy160.

Krzywinski M et al. 2009. Circos: An information aesthetic for comparative genomics. Genome Res. 19:1639–1645. doi: 10.1101/gr.092759.109.

Laloi D, Richard M, Lecomte J, Massot M, Clobert J. 2004. Multiple paternity in clutches of common lizard *Lacerta vivipara*: Data from microsatellite markers. Mol. Ecol. 13:719–723. doi: 10.1046/j.1365-294X.2004.02102.x.

Li H et al. 2009. The Sequence Alignment/Map format and SAMtools. Bioinfomatics. 25:2078–2079. doi: 10.1093/bioinformatics/btp352.

Li H, Durbin R. 2009. Fast and accurate short read alignment with Burrows-Wheeler transform. Bioinfomatics. 25:1754–1760. doi: 10.1093/bioinformatics/btp324.

Lind AL et al. 2019. Genome of the Komodo dragon reveals adaptations in the cardiovascular and chemosensory systems of monitor lizards. Nat. Ecol. Evol. 3:1241–1252. doi: 10.1038/s41559-019-0945-8.

Liu Y et al. 2015. *Gekko japonicus* genome reveals evolution of adhesive toe pads and tail regeneration. Nat. Commun. 6:10033. doi: 10.1038/ncomms10033.

Löytynoja A, Goldman N. 2010. WebPRANK: A phylogeny-aware multiple sequence aligner with interactive alignment browser. BMC Bioinformatics. 11:579. doi: 10.1186/1471-2105-11-579.

Morgulis A, Gertz EM, Schäffer AA, Agarwala R. 2006. WindowMasker: Window-based masker for sequenced genomes. Bioinfomatics. 22:134–141. doi: 10.1093/bioinformatics/bti774.

Niknafs YS, Pandian B, Iyer HK, Chinnaiyan AM, Iyer MK. 2016. TACO produces robust multisample transcriptome assemblies from RNA-seq. Nat. Methods. 14:68–70. doi: 10.1038/nmeth.4078.

Pertea M et al. 2015. StringTie enables improved reconstruction of a transcriptome from RNA-seq reads. Nat. Biotechnol. 33:290. doi: 10.1038/nbt.3122.

Peterson BK et al, Weber JN, Kay EH, Fisher HS, Hoekstra HE. 2012. Double digest RADseq: an inexpensive method for *de novo* SNP discovery and genotyping in model and non-model species. PLoS One. 7:1–11. doi: 10.1371/journal.pone.0037135.

Ponsting H, Ning Z. 2010. SMALT - A New Mapper for DNA Sequencing Reads. F1000 Posters. 1:(L313). doi: 10.7490/F1000RESEARCH.327.1.

Pruitt KD, Tatusova T, Maglott DR. 2007. NCBI reference sequences (RefSeq): A curated non-redundant sequence database of genomes, transcripts and proteins. Nucleic Acids Res. 35:D61–D65. doi: 10.1093/nar/gkl842.

Quinlan AR. 2014. BEDTools: The Swiss-Army tool for genome feature analysis. Curr. Protoc. Bioinforma. 47:11–12. doi: 10.1002/0471250953.bi1112s47.

Recknagel H, Jacobs A, Herzyk P, Elmer KR. 2015. Double-digest RAD sequencing using Ion Proton semiconductor platform (ddRADseq-ion) with nonmodel organisms. Mol. Ecol. Resour. 15:1316–1329. doi: 10.1111/1755-0998.12406.

Recknagel H, Kamenos NA, Elmer KR. 2018. Common lizards break Dollo’s law of irreversibility: Genome-wide phylogenomics support a single origin of viviparity and re-evolution of oviparity. Mol. Phylogenet. Evol. 127:579–588. doi: 10.1016/J.YMPEV.2018.05.029.

Simão FA, Waterhouse RM, Ioannidis P, Kriventseva E V, Zdobnov EM. 2015. BUSCO: assessing genome assembly and annotation completeness with single-copy orthologs. Bioinfomatics. 31:3210–3212. doi: 10.1093/bioinformatics/btv351.

Song B et al. 2015. A genome draft of the legless anguid lizard, *Ophisaurus gracilis*. Gigascience. doi: 10.1186/s13742-015-0056-7.

Stamatakis A. 2014. RAxML version 8: a tool for phylogenetic analysis and post-analysis of large phylogenies. Bioinfomatics. 30:1312–1313. doi: 10.1093/bioinformatics/btu033.

Stanke M et al. 2006. AUGUSTUS: ab initio prediction of alternative transcripts. Nucleic Acids Res. 34:W435–W439. doi: 10.1093/nar/gkl200.

Tang H et al. 2015. ALLMAPS: Robust scaffold ordering based on multiple maps. Genome Biol. 16:3. doi: 10.1186/s13059-014-0573-1.

Tange O. 2011. GNU Parallel: the command-line power tool. USENIX Mag. 36:42–47. doi: 10.5281/zenodo.16303.

Uller T, Olsson M. 2008. Multiple paternity in reptiles: Patterns and processes. Mol. Ecol. 17:2566–2580. doi: 10.1111/j.1365-294X.2008.03772.x.

Vonk FJ et al. 2013. The king cobra genome reveals dynamic gene evolution and adaptation in the snake venom system. Proc. Natl. Acad. Sci. U. S. A. 110:20651–20656. doi: 10.1073/pnas.1314702110.

Xiong Z et al. 2016. Draft genome of the leopard gecko, *Eublepharis macularius*. Gigascience. 5:s13742-16. doi: 10.1186/s13742-016-0151-4.

Yin W et al. 2016. Evolutionary trajectories of snake genes and genomes revealed by comparative analyses of five-pacer viper. Nat. Commun. 7:13107. doi: 10.1038/ncomms13107.
